# The Rise of State Preventive Medicine

**Published:** 1917-01-20

**Authors:** 


### PUBLIC HEALTH ADMINISTRATION.*

### The Rise of State Preventive Medicine.

Public health administration is a comparatively modern development in our local government system. As Mr. Bannington shows, it was not until the middle of the last century that local authorities were constituted and empowered to deal in any way with the protection and promotion of the public health. Until that time, individualism in this as in other things was rampant, and each citizen was left free to do what he liked with his own dwelling-house and its sanitation; there was little or no interference in the general interest with such matters as water supply, removal of refuse, etc. As we all know now the results were most deplorable, " one out of every thirty of the population of towns dying every year," whilst, of course, in years of epidemic the mortality was very much higher. It was the severity of the cholera epidemic which stimulated Parliament to action and led in 1848 to the passing of the first Public Health Act, " the groundwork of our sanitary legislation," creating local boards of health either on petition by the ratepayers or compulsorily when the death-rate reached 23 per 1,000," whose duty it was to appoint inspectors of nuisances and—if they thought fit—medical officers of health. This Act needed much strengthening by subsequent enactments—in 1866, 1872, 1874-5—before its provisions could be made effective throughout the country. In every decade since this date fresh legislation has been passed with the object of improving and reinforcing the system of public health administration thus created, until we have at work to-day that great power for hygienic righteousness whose varied activities Mr. Bannington describes and explains with a care and lucidity which cannot be too highly commended.

Mr. Bannington defines the authorities and the areas of their jurisdiction, explains the local and public legislation from which they derive their powers, gives a most informing account of the administrative organisation and discriminates with knowledge and impartiality between the parts taken, in a well-managed authority, by the officials and elected members respectively. In separate chapters the author deals fully with the duties and powers of medical officers and sanitary inspectors. We learn that between 1873 and 1911 the number of whole-time appointments of medical officers of health rose from 249 to 501, the percentage of the population concerned being 20.6 in the former and 61.4 in the latter year. In 1914 there were 1,986 inspectors of nuisances, of whom only 983, however, gave their whole time to the office. The total number, Mr. Bannington points out, could probably be reduced with advantage, if more of the smaller authorities availed themselves of the permission of the Local Government Board to combine with one another for the appointment of whole-time officers. Mr. Bannington refers to the recent appointment of women inspectors and health visitors, and is of the opinion, contrary to the view prevalent in some quarters, that there should be no clashing between the two classes of officials, their duties, in his judgment, being capable of clear demarcation.

In his chapter on " Hospitals and Hospital Administration " Mr. Bannington clearly shows how, in the nature of things, local authorities have been driven to take up the responsibility of providing accommodation for the sick, both as regards sufferers from infectious as well as other diseases and from accidents. In fact, Mr. Bannington regrets that " there is no general obligation upon local authorities to provide treatment or accommodation for any particular sick person, however needy he may be, or however dangerous it is to the public for him to remain at home without proper care, treatment, or isolation "—a view which appears to call for more exposition than the author gives it.

Mr. Bannington discusses other topics to which we should like to refer but for the exigencies of space. We must reserve a word or two, however, for his final chapter on " The Need for Reform." Mr. Bannington shows that, judged by the statistical test, our system of public health administration has splendidly vindicated itself. But he is not content with this success. He believes that by simplifying, codifying, and in some respects amending the present law still better results may be secured, and we cannot say that he is wrong.

* *English Public Health Administration.* By B. G. Bannington. London : P. S. King and Son. 7s. 6d. net.

